# Lymphoepithelial Carcinoma of Larynx and Hypopharynx: A Rare Clinicopathological Entity

**DOI:** 10.3390/cancers12092431

**Published:** 2020-08-27

**Authors:** Muhammad Faisal, Sabrina Hartenbach, Annemarie Schratter, Wolfgang J. Köstler, Hannes Kaufmann, Rudolf Seemann, Claudia Lill, Sasan Hamzavi, Arno Wutzl, Boban M. Erovic

**Affiliations:** 1 Institute of Head and Neck Diseases, Evangelical Hospital, 1180 Vienna, Austria; maxfas@live.com (M.F.); rudolf.seemann@gmail.com (R.S.); c.lill@ekhwien.at (C.L.); sasanhamzavi@me.com (S.H.); arno.wutzl@meduniwien.ac.at (A.W.); 2 Department of Surgical Oncology, Shaukat Khanum Memorial Cancer Hospital and Research Center, Lahore 54000, Pakistan; sabrina.hartenbach@icloud.com; 3 Institute of Radio-Oncology, Kaiser-Franz-Josef Hospital, 1180 Vienna, Austria; annemarie.schratter@wienkav.at; 4 Clinical Division of Oncology, Department of Medicine, Comprehensive Cancer Center, Medical University of Vienna, 1180 Vienna, Austria; w.koestler@imed19.at; 5 Clinical Oncology and Hematology, Kaiser-Franz-Josef Hospital, 1180 Vienna, Austria; hannes.kaufmann@wienkav.at

**Keywords:** head and neck neoplasms, larynx, hypopharynx, Epstein–Barr virus, human papillomavirus

## Abstract

(1) Background: Lymphoepithelial carcinoma of the hypopharynx and larynx is a rare tumor with fewer than 50 cases in the published literature. We present a literature review to discuss the clinical findings, viral or genetic associations, diagnostic challenges, histopathological findings and therapeutic aspects of the disease. (2) Methods: A comprehensive literature review was performed through MEDLINE/PubMed from 1968 to 2018. We identified 21 studies comprising 46 patients. Data on all the clinicopathological features, diagnostic modalities, treatment options and viral or genetic etiology were extracted and analyzed using SPSS. (3) Results: The mean age of presentation was 64 years (range 40–82 years) and mostly involved males. The supraglottis and pyriform sinus were the most commonly involved sub-sites, with surgery as the preferred treatment modality. The presence of the Epstein–Barr virus possibly directs a viral etiology. The incidence of cervical and distant metastasis was 54% and 21%, respectively. The median survival time was 30 months. (4) Conclusions: Lymphoepithelial carcinoma of the hypopharynx is an aggressive tumor with a strong predilection for regional and distant metastasis. Surgery, in combination with adjuvant therapy, provides promising results. Immunohistochemistry helps in differentiating LEC from other pathologies.

## 1. Introduction

Lymphoepithelial carcinoma (LEC) of the larynx and hypopharynx is a rare neoplasm with a close association with the Epstein–Barr virus (EBV) and a high incidence of regional and distant metastasis at the time of presentation [1]. The disease has a strong propensity to occur in Caucasian males in their fifth to seventh decades of life, unlike its nasopharyngeal counterpart, which is more prevalent in the Chinese population. The oropharynx, laryngopharynx, trachea, salivary glands, sinonasal tract and oral cavity are reported as possible sites of involvement in the head and neck. Clinically, the most common symptoms are either dysphagia or dysphonia, which is related to the locality of the tumor [2]. Smoking and alcohol are thought to be contributing factors, with no clear association with the Epstein–Barr virus as in nasopharyngeal LEC [3]. Recent literature has revealed a link between oropharyngeal LEC and human papillomavirus (HPV) in 86–94% of cases, along with p16 and p53 overexpression and a survival outcome similar to that of other HPV-related oropharyngeal tumors [4,5]. LECs are thought to exist in two forms: a pure LEC tumor and a hybrid form containing both LEC and squamous cell carcinoma (SCC) components. This fact was also noticed in both primary and metastatic deposits. Surgery has remained an integral part of the treatment for lymphoepithelial carcinomas in the past, along with radiotherapy owing to the radiosensitive nature of the tumor. Recently, the role of adjuvant chemotherapy has been considered in the wake of early regional and distant metastasis [6]. We report a case of lymphoepithelial carcinoma along with a comprehensive review of the literature to discuss the clinical trends, viral and genetic associations, diagnostic challenges, histopathological findings and the therapeutic aspects of this rare entity.

## 2. Materials and Methods

The literature search was performed using MEDLINE/PubMed for words such as “Lymphoepithelial carcinoma of hypopharynx”, “Lymphoepithelial carcinoma of larynx”, “LEC, hypopharynx”, “LEC, larynx” and “Non-nasopharyngeal undifferentiated carcinoma”. Inclusion criteria were case reports, retrospective series and histological confirmation of LEC of the hypopharynx or larynx, including all sub-sites. Exclusion criteria were lymphoepithelial carcinoma of other sites. The result of the search was 36 studies, of which 8 were excluded due to descriptions of different histologies, while 7 were removed due to the presence of LECs in sites other than the larynx or hypopharynx. After a comprehensive review, 21 articles were identified in the English literature from 1968 to 2018 [1,2,6,7,8,9,10,11,12,13,14,15,16,17,18,19,20,21]. Clinical information, including demographics, tumor location (sub-site), presenting symptoms, treatment modalities, viral etiology, genetic mutations and survival status, were extracted (Table 1). Additionally, a case of LEC presented and treated at the Institute of Head and Neck Diseases was also included. Ethical approval was granted by the Ethics Committee, Evangelisches Krankenhaus, Vienna, and the patient’s identification or confidentiality has not been breached. SPSS version 24 (IBM, Armonk, New York, NY, USA) was used for the statistical analyses. Kaplan–Meier analysis was performed to determine the OS and DSS along with the impact of potential variables on DSS. The corresponding *p*-values were obtained via the log-rank test.

## 3. Results

**Figure 1:**
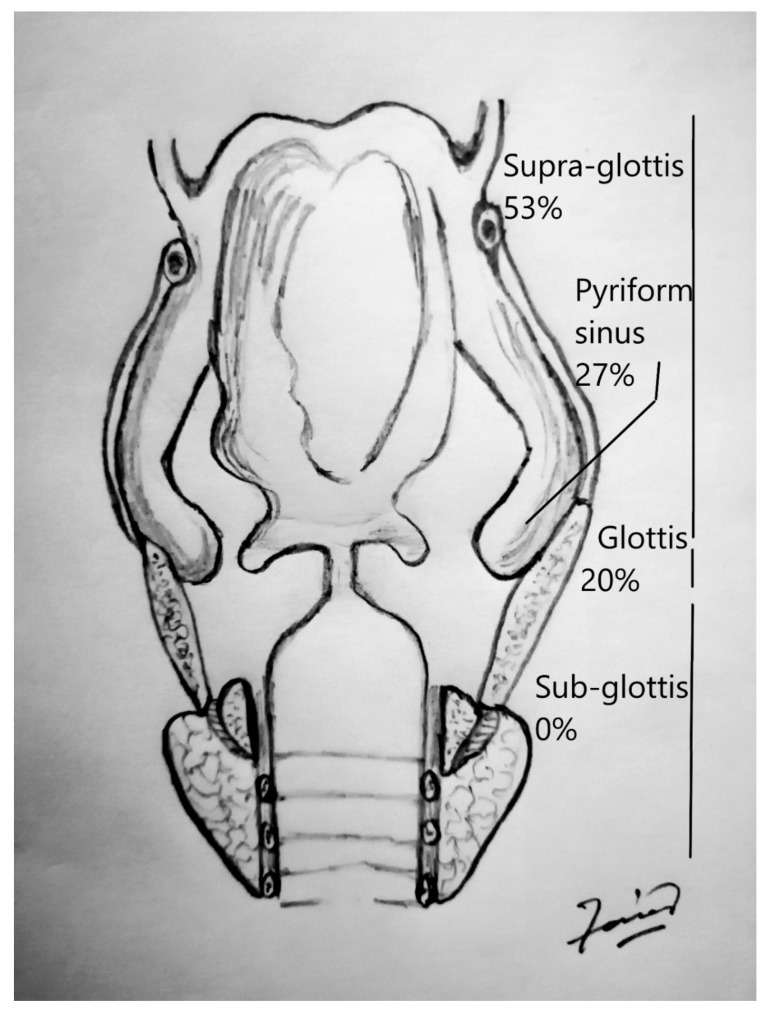
Distribution of lymphoepithelial carcinoma (LEC) in laryngeal and hypopharyngeal sub-sites.

**Figure 2:**
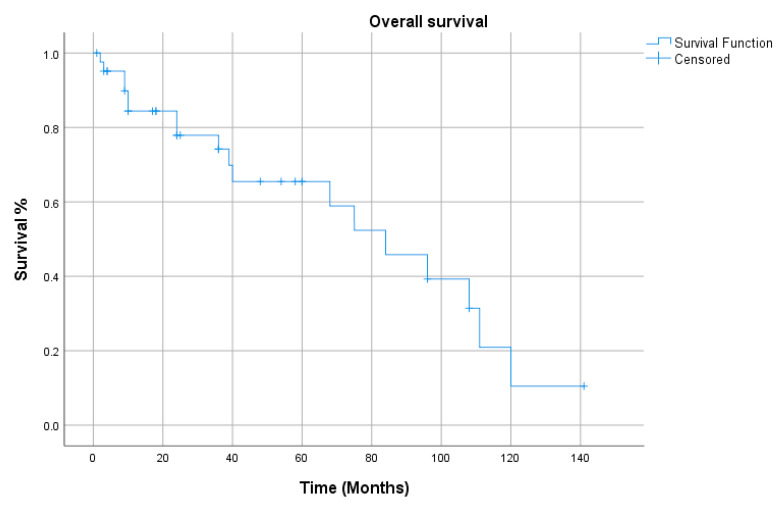
Overall survival.

**Figure 3:**
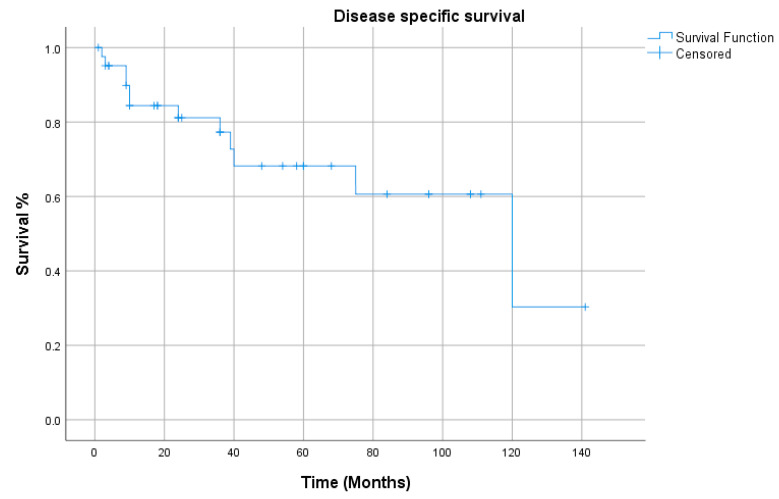
Disease-specific survival.

**Figure 4:**
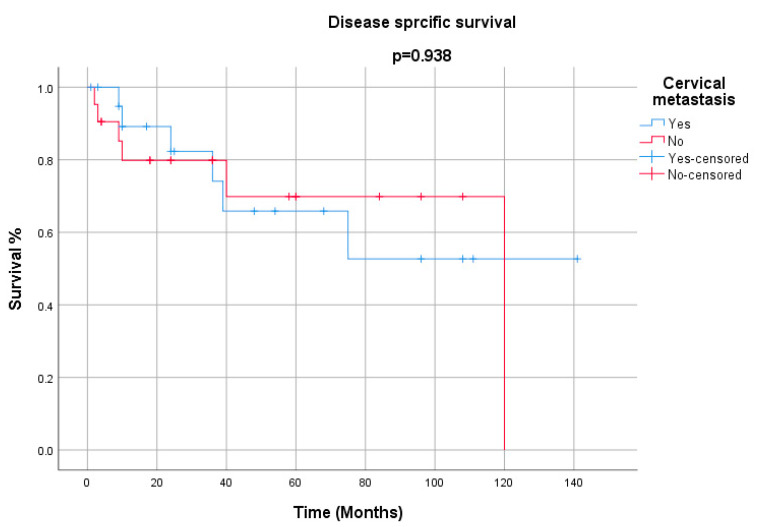
Disease-specific survival for cervical metastasis.

**Figure 5:**
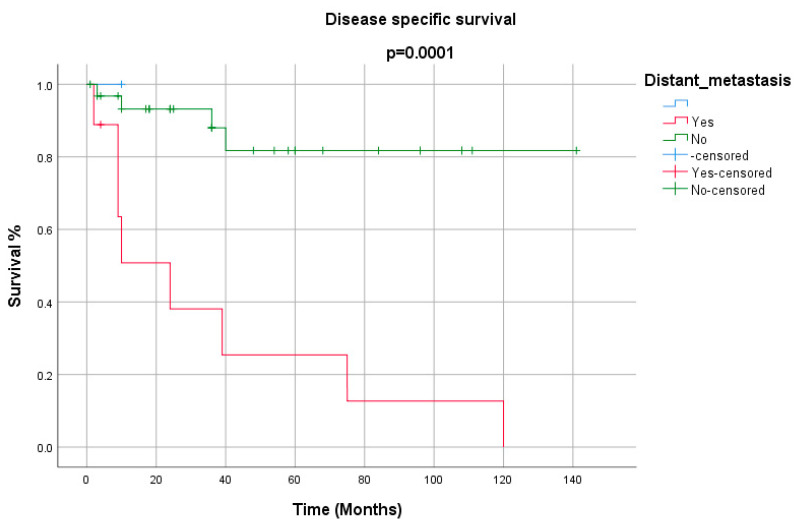
Disease-specific survival for distant metastasis.

**Figure 6:**
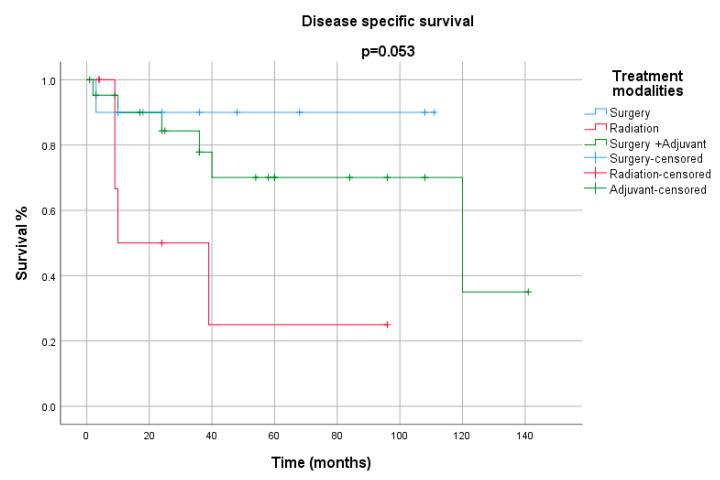
Disease-specific survival for treatment modalities.

All the demographics and clinical characteristics for the studies included in the comprehensive review are presented in Table 1 and Table 2. The majority of patients were males (91%) with a median age of 64 years (range 40–82 years). The supraglottis was the most common sub-site of involvement among laryngeal LECs, while the pyriform sinus was reported as the most frequent location among hypopharyngeal tumors (Figure 1). The most common presenting complaints were hoarseness (37%), dysphagia (22%) and cervical mass (22%). Surgery, either alone (26%) or in combination (53%), has remained the main treatment modality. The median follow-up was 36 months. During their last follow-up, 24 patients were alive, while 11 had died of a recurrence of the disease, and 6 patients succumbed to other medical conditions. Only 4 patients had been lost to follow-up. Metastasis to cervical lymph nodes, either at the time of presentation or later in the course of follow-up, was observed in 54% of patients, while 21% had developed distant metastasis. The overall 5-year and disease-free survival (DFS) rates were 65% and 68%, respectively (Figure 2 and Figure 3). The mean and median survival times were 42 and 30 months. The DFS for node-negative and node-positive patients was 69% and 65%, respectively (*p* = 0.938). Contrary to that, distant metastasis significantly impacted DFS (81% vs. 25%, *p* = 0.0001). Among the treatment modalities, surgery alone had the best outcome in terms of disease-free survival as compared to radiation alone or surgery in combination with adjuvant (90% vs. 70% vs. 25%). Kaplan–Meier survival analysis showed that no distant metastasis (*p* = 0.0001) and surgery as the treatment modality (*p* = 0.053) were the prognosticators of improved survival in the cumulative analysis (Figure 4, Figure 5 and Figure 6).

We also present a case of a 63-year-old male who presented with the complaint of dysphagia, along with a tender and immobile right neck mass measuring 5 cm at the vertical extension, in the Institute of Head and Neck Diseases, Evangelisches Krankenhaus, Vienna. The flexible nasopharyngoscopy showed a paralytic right vocal cord with an exophytic mass involving the right pyriform sinus. Imaging showed a hypopharyngeal tumor (T4 N3 M0) with no distant metastasis. The tumor mass had occupied the entire pyriform sinus on the right side, with a vertical extension of 2–3 cm. The apex of the pyriform sinus was clear and the right larynx was fixed. The scan showed a 6 cm fixed ipsilateral cervical lymph node.

The surgical procedure comprised a laryngo-pharyngectomy, bilateral neck dissection and pectoralis major flap reconstruction. The specimen measured 9 × 7 × 5 cm in its greatest dimension and showed a supraglottic tumor (3 × 2 cm) on the right side with protrusion through the mucous membrane. The remaining laryngeal mucosa and resected portion of the trachea remained unremarkable. The final histopathology showed LEC with trans-glottic extension (pT4 aN3 b), along with perineural and lympho-vascular invasion. Out of 48 lymph nodes retrieved, 9 were positive for lymphoepithelial carcinoma (bilateral involvement) along with extra-capsular extension (ECE). The primary specimen showed positivity for the Epstein–Barr virus (EBV), but immunohistochemistry for p16 was negative. All the resected surgical margins were free of the tumor.

**Figure 7:**
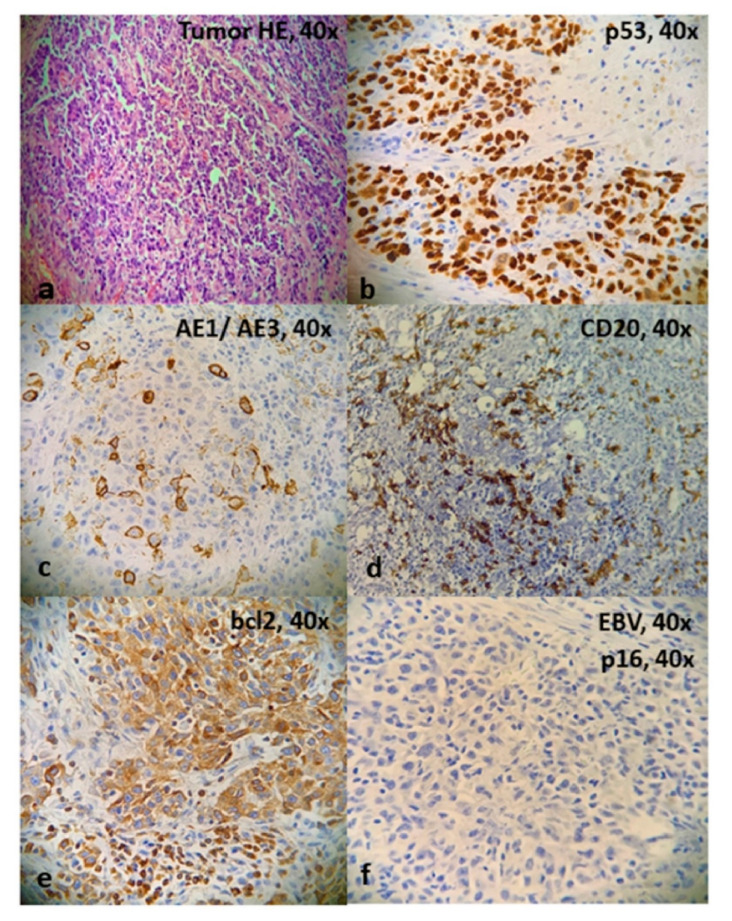
(**a**) Cells present in solid nests with pleomorphic appearance. Clear cell nuclei are seen with prominent nucleoli. (**b**) p53 association with >80% mutated cells. (**c**) Immunohistochemistry was positive for AE1/AE3. (**d**) Immunohistochemistry was negative for CD20. (**e**) Strong staining for bcl-2. (**f**) No evidence of p16 was found with immunochemistry.

**Figure 8:**
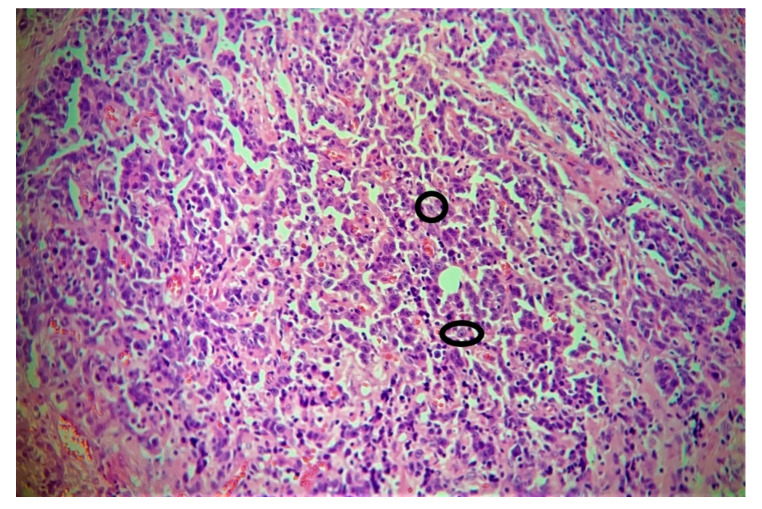
Clear cell nuclei, along with prominent nucleoli, are demonstrated. Dense lymphocytic infiltration is also seen in the background.

The homogeneous yellowish-white tumor had penetrated through the thyroid cartilage into adjacent soft tissue. The laryngeal skeleton showed central ossification closed to the hyaline cartilage. Adjacent to the ventral side, there were striated skeletal muscle fibers with tumor infiltrates. The tumor cells were present in solid nests with a pleomorphic appearance. Clear cell nuclei, along with prominent nucleoli, were demonstrated. Dense lymphocytic infiltration was also seen with a special affinity for nerve sheaths (Figure 7a–f and Figure 8). The tumor showed trans-glottic spread and penetration through the ventral laryngeal skeleton with invasion of the attached musculature. The largest lymph node removed was completely involved by the tumor, with a non-keratinizing lymphoepithelial carcinoma that had solid growth. The cellular nuclei were highly pleomorphic and showed atypical mitosis and comedo-like necrosis with large central sclerotic zones and hemorrhages. The tumor infiltrates showed large-scale capsule sprouting growth, with perineural sheath infiltration as well as ingrowth into the attached striated skeletal muscle. Individual tumor cells were also found in vessel lumen. Immunohistochemistry showed positivity for Pan-keratin (AE1/3) and the nuclear expression of p63 while remaining negative for CK7, Synaptophysin, Chromogranin A, S-100, TTF1, CD20, PDL1 and p16. The tumor showed a strong expression of p53 (> 80% mutated cells). The presence of EBV was confirmed by a molecular biological examination, where DNA was extracted from the tumor tissue. The proof of EBV by means of pathogen-specific PCR (AB Analitica, proven positive internal control) was based on the following result: amplification was detected and positive for the Epstein–Barr virus (EBV) (Figure 2). The Multi-disciplinary Tumor Board (MTB) advised adjuvant chemoradiotherapy. A total dose of 66 Grey (Gy), 2 Gy/day, was scheduled and started simultaneously with chemotherapy. Radiotherapy treatment was performed by using volumetric modulated arc therapy (VMAT). Concurrent chemotherapy consisted of weekly cisplatin at a dose of 40 mg/m^2^. Upon the completion of therapy after 4 weeks, cisplatin-related renal toxicity was observed. Consequently, the protocol was changed to carboplatin weekly, and chemotherapy was continued for an additional 3 weeks. The radiation field also included neck nodal levels bilaterally, and a total dose of 56 Gy was delivered. The daily fraction was 2 Gy to the tumor bed and 1.7 Gy to the neck nodes, resulting in five fractions per week. Accuracy of treatment was ensured by using daily image-guided and adaptive radiotherapy (cone-beam CT).

## 4. Discussion

The World Health Organization (WHO) has defined LEC as “a poorly differentiated squamous cell carcinoma or histologically undifferentiated carcinoma accompanied by a prominent reactive lymphoplasmacytic infiltrate, morphologically similar to nasopharyngeal carcinoma’’. Most of the literature published is either in the form of case reports or series. The history dates back to 1921 when Schmincke in Germany, as well as Regaud and Reverchon in France, coined the term [22,23]. Marx was the first person to report a case of lymphoepithelial carcinoma of the pyriform sinus involving the larynx in 1926 [24]. Calvet and Ferlito, in a large review of 2052 laryngeal cancer cases, documented a rare LEC of the larynx [8,25]. Before 1980, case reports addressing LECs had been published under terminologies like “Lymphoepithelial carcinoma Schmincke Regaud”. A variety of other names have also been attributed to this tumor category, such as undifferentiated carcinoma of nasopharyngeal type, undifferentiated carcinoma with lymphoid stroma, lymphoepithelioma and lymphoepithelial like carcinoma, as well as recent names like lymphoepithelial carcinoma (LEC). Recently, Acuna, Hammas and Kermani have highlighted the etiopathogenesis of the disease in the background of viral etiology [11,12,13].

LEC is a rare but aggressive entity. Ferlito has been able to identify only 1 case (0.05%) in a series of 2052 cases of hypopharyngeal and laryngeal carcinomas [8]. Acuna has postulated an even lower actual incidence (0.06–0.2%). Micheau, MacMillan, Zbaren and Acuna have reported male predominance in their respective series [6,11,14,15]. In our cumulative series, the disease predominates in males (76%), with smoking and alcohol as major contributing factors. Although smoking and alcohol have been mentioned in some of the published case reports and series, the cause–effect relationship was never established [1,12,16]. Andryk and Bansal have also failed to establish such a link. It is postulated that smoking may play more of a role in nasopharyngeal LEC than in non-nasopharyngeal counterparts [10,18]. The etiology of laryngeal or hypopharyngeal lymphoepithelial carcinoma still seems to be unclear.

Controversial opinions exist regarding the association between LEC and EBV. EBV infections are more closely associated with the LECs of salivary glands, lungs, thymus and stomach [2]. The association of EBV with LECs and Nasopharyngeal carcinomas (NPCs) has been reported more in Southeast Asia, but there have been conflicting reports in the West. MacMillan has studied eight cases of LECs of the larynx but could not find EBV in any of them. He has concluded that the EBV association with LEC is attributed to individuals of non-Asian origin [6]. Acuna has reported 6 out of 19 cases of LEC of the larynx with EBV positivity, contrary to its nasopharyngeal counterpart with a strong EBV association. Acuna has found HPV DNA to be positive in 50% of cases (four of type 16 and one of type 58) but none in hypopharyngeal carcinoma [11]. Our cumulative results have shown 20% of cases to be EBV related. LEC has also been associated with p53 gene mutations, with studies by Macmillan and Acuna reporting high numbers of mutated p53 cases and advocating genetic aberrations to be the causative factors. Macmillan et al. have found the high rate of p53 damage in LEC to be consistent with squamous cell carcinoma (SCC), where p53 mutations have been reported to be an early event in the tumor process [6]. This has resulted in further classifying these tumors as either p16-positive/p53-negative (viral etiology) or p16-negative/p53-positive (non-viral, genetic etiology). The case series by Acuna has highlighted the involvement of HPV, with overexpressed p16 in three cases, HPV without p16 overexpression in one case and HPV 58 in one case. The presence of high-risk HPV varies among different sites, with the highest number of cases being reported in the oropharynx (86–94%) [4,5]. Similarly, more than 30% of nasopharyngeal carcinomas harbor HPV. The most common presenting complaints reported in the literature for hypopharyngeal or laryngeal LEC were dysphagia, hoarseness and cervical mass. The patients presented to clinicians after the emergence of the above-mentioned symptoms. The most common sub-site of involvement was the pyriform sinus in hypopharyngeal LECs [6,11]. Within the larynx, the supraglottis was affected more than other sub-sites [1].

The modes of invasion of laryngeal and pharyngeal carcinomas have also been elaborated by Micheau et al. LECs have traditionally been associated with laryngoceles. Histological evaluations have shown squamous or cylindrical epithelium with organized lymphoid tissue similar in histology to lymphoid tissue or lymph nodes of Waldeyer’s ring. These laryngoceles were also postulated to be true tonsils. These lymphoid-like structures have been thought of as the source or origin of tumors in these sites [14].

The operative and biopsied samples of LEC are difficult to distinguish from malignant melanomas and non-Hodgkin’s lymphoma. Cytokeratin expression by tumor cells helps to distinguish LEC from these tumors using immunohistochemistry. Immunomarkers, such as chromogranian and synaptophysin, Melan A and HMB45 or desmin and smooth muscle actin, are applied to differentiate neuroendocrine, melanoma and smooth muscle tumors, respectively. Histological appearances depict clumps of large undifferentiated cells with indistinct cell borders, intermingled with a dense inflammatory infiltrate composed of lymphocytes and plasma cells [1,12,26]. The histological picture resembles that of non-keratinizing nasopharyngeal carcinomas [27]. Approximately half of the cases diagnosed with LEC have shown a component of squamous cell carcinoma [28].

Surgery and radiotherapy have constituted the major part of published series in terms of the management of laryngeal and hypopharyngeal LECs. Historically, treatment strategies have remained controversial for LECs. Stanley preferred upfront radiotherapy in his cases, with recurrences occurring in all except one [29]. Micheau et al. supplemented surgery with post-operative radiotherapy, and only one died of the disease in 3 years of follow-up [14]. Macmillan has reported the rate of occult metastasis to be significantly high (88%) and used surgery as the primary treatment modality with radiation as an adjuvant, and none of the patients died of disease recurrence or distant metastasis, while one patient was lost to follow-up [6]. Laryngeal LECs are radiosensitive tumors with good control rates once subjected to radiotherapy [12,17]. Marioni has described a 75% risk of nodal metastasis, with 25% of patients having a disseminated disease at the time of presentation [1]. Keeping in view the poor outcome owing to distant metastasis, the role of induction chemotherapy has still remained controversial. Kermani used neoadjuvant chemotherapy with a response rate of 30% at the primary site and 50% at regional nodes [13]. Neo-adjuvant chemotherapy has been recommended in a few studies to reduce the disease volume in a clinically positive lymph node and to decrease the risk of distant failures [19]. Lymph node involvement by LECs, either at presentation or later in the course of follow-up, has shown no significant difference in disease-free survival. The same trend was noticed by Chan et al. in non-nasopharyngeal LECs.

In our cumulative data, most of the patients were treated with either surgery alone (26%) or in combination with radiotherapy (37%) or chemoradiotherapy (15%). Among those treated with surgery, only one died of the disease, one was lost to follow-up and three died of other causes. The probable reason for the improved outcome (90%) in the surgery-only group was the early stage of the disease in these patients. For advanced cases, adjuvant modalities were used for obvious reasons. Thus, disease stage may be a confounding factor in the selection of single or multiple treatment modalities. When surgery was used in combination with radiotherapy, only one patient out of four died of the disease, while three were alive with no recurrence. Chan et al. have reported disease-specific mortality of 60% in SEER data, but Acuna published better 5-year overall and disease-specific survival (60% and 100%), with a second neoplasm considered to be the main cause of mortality [30]. Future studies will surely elaborate on the role of induction chemotherapy, particularly in cases presenting with metastatic neck disease, to minimize the risk of distant metastasis. One possible explanation for the high incidence of second malignancies was probably the unusually long follow-ups of these patients, as mentioned by Acuna et al. [11].

In the supraglottic larynx, the differential diagnosis of LEC includes laryngeal large cell neuroendocrine carcinoma (LCNEC), which is a recently identified sub-type of poorly differentiated neuroendocrine tumors. It is of dire importance to differentiate LCNEC from LEC, as the treatment and prognosis differ markedly. The treatment for LCNEC mostly includes chemoradiotherapy [31,32].

Of course, this study has its limitations and biases that must be considered. Despite the inclusion of only laryngeal and hypopharyngeal carcinomas, a heterogeneous patient population cannot be avoided, as the case reports come from different countries and continents. In addition, there may be differences in the quality of the examination or treatment, treatment protocol and follow-up care between the clinical centers (performance bias). A publication bias cannot be ruled out, as case reports occasionally describe unusual events or therapeutic strategies that may deviate from the norm. Potential outcome-relevant factors such as the extent of the surgical margin could not always be ascertained in included case reports, as the authors usually only described it as R0 resection. Inappropriate case reports were excluded as a priority to minimize the limitations and bias of the study. Furthermore, two independent authors collected data to avoid reader bias.

## 5. Conclusions

Lymphoepithelial carcinomas of the hypopharynx are rare, aggressive neoplasms with a low incidence and prevalence, along with high risk of occult metastasis and distant spread. The incidence of cervical and distant metastasis in our cumulative data was 55% and 18%, respectively. Such a high risk of cervical metastasis justifies elective neck treatment in these patients. These tumors have shown better survival outcomes in recent years when surgery was used in combination with adjuvant therapy. Distant metastasis has proved to be a predictor of poor survival. Immunohistochemical markers help to distinguish LEC from other histologies that are similar in appearance. The association with viral histology is yet to be established.

## Figures and Tables

**Table 1:** Patient characteristics.

Variables	Number of Patients N (%)
**Gender**	
Male	41(91)
Female	04(9)
**Site**	
**Larynx**	
–Supraglottis	24
–Glottis	9
–Subglottis	0
**Hypopharynx**	
–Pyriform sinus	12
**Associated factors**	
Smoking	12
Alcohol	4
Not reported	28
**Symptoms**	
Hoarseness	17
Dysphagia	10
Neck mass	10
Dyspnea	2
Odynophagia	1
Others (Globus sensation, vomiting, sore throat)	5
**Treatment**	
Surgery	12
RT	9
Surgery + Radiation	17
Surgery + Chemoradiotherapy	7
**Cervical metastasis**	
Yes	24
No	20
Not reported	1
**Distant metastasis**	
Yes	9
No	32
Not reported	4
**EBV**	
Positive	5
Negative	24
Not reported	16
**HPV**	
Positive	4
Negative	4
Not reported	37
**P53**	
Positive	10
Negative	4
Not reported	31

Abbreviations: RT, radiotherapy; EBV, Epstein–Barr virus; HPV, human papillomavirus.

**Table 2:** Demographics and clinical characteristics of the studies included in the review.

Author	Year	Age	Gender	Site	Smoking	Alcohol	Symptoms	Follow
								up
Dockerty	1968	69	M	Hypopharynx	–	–		10
Ferlito	1977	60	M	Pyriform sinus	–	–	Neck mass	10
Toker	1978	69	F	Glottis	–	–	Hoarseness	108
Micheau	1979	40	M	Epiglottis	–	–	Dysphagia, otalgia	54
		62	M	Epiglottis	–	–	Dysphagia, Hoarseness	2
		68	M	Glottis	–	–	Neck mass	60
Stanley	1985	52	M	Glottis	–	–	Dyspnea	9
		50	F	Supraglottis	–	–		75
		63	M	Supraglottis	–	–	Neck mass	39
		53	F	Supraglottis	–	–	Dysphagia/Hoarseness	96
Navarrete	1989	64	M	Glottis	–	–		36
Frank	1995	67	M	Pyriform	–	–		NA
				Sinus				
Narozny	1995	59	M	Supraglottis	–	–	Hoarseness, Cervical mass	24
Andryk	1996	60	M	Pyriform sinus	–	–	Dysphagia/	4
							Cough	
MacMillan	1996	77	M	Pyriform sinus	Yes	–	Hoarseness	58
		54	M	Pyriform sinus	–	Yes	Sore throat	10
		59	M	Pyriform sinus	–	–	Hoarseness	3
		59	M	Pyriform sinus	–	–	Hoarseness/	1
							Neck mass/	
							Dysphagia	
		75	M	Glottis	Yes	Yes		Lost
		53	F	Epiglottis	Yes	Yes		25
		51	M	Epiglottis	Yes			17
		82	M	Glottis	Yes			10
Zbaren	1997	53	M	Glottis	NR	NR	Hoarseness, Neck mass	120
		71	M	Pyriform fossa	NR	NR	Dysphagia, Neck mass	36
		66	M	Supraglottis	NR	NR	Hoarseness, Neck mass	60
		73	M	Glottis	NR	NR	Hoarseness	36
Dray	1998	70	M	Supraglottis	Yes	–	Globus sensation	36
Sone	1998	71	M	Supraglottis			Dysphagia/Hoarseness	3
Marioni	2002	67	M	Supraglottis	Yes	–	Hoarseness	48
Coskun	2005	60	M	Epiglottis	Yes	–	Neck mass	24
Bansal	2011	60	M	Supraglottis	Yes	Yes	Hoarseness, dysphagia, Odynophagia	4
Ibrahimov	2013	58	M	Glottis	No	No	Hoarseness	18
Kermani	2015	73	M	Glottis	–	–	Dysphonea, Dyspnea	18
Asma	2017	81	M	Glottis, Supraglottis	Yes	No	Dysphonia, Dyspnea, Dysphagia	9
Hammas	2017	81	M	Supraglottic	Yes	No	Dysphonia, Dyspnea, Dysphagia	–
				Glottic				
				Subglottic				
Acuna	2018	68	M	Pyriform sinus			NR	24
		71	M	Pyriform sinus			NR	111
		80	M	Pyriform sinus			NR	68
		76	M	Supraglottis			NR	40
		58	M	Glottis			NR	NA
		49	M	Supraglottis			NR	24
		46	M	Supraglottis			NR	141
		53	M	Supraglottis			NR	96
		74	M	Glottis			NR	84
		65	M	Supraglottis			NR	108
Monteiro	2019	59	M	Supraglottis	Yes	No	Odynophagia, dysphonia	9
